# Intra-and interobserver reliability of determining the femoral footprint of the torn anterior cruciate ligament on MRI scans

**DOI:** 10.1186/s12891-021-04376-5

**Published:** 2021-05-28

**Authors:** M. J. M. Zee, R. A. Sulaihem, R. L. Diercks, I. van den Akker-Scheek

**Affiliations:** Department of Orthopedic Surgery, University of Groningen, University Medical Center Groningen, PO Box 30.001, 9700RB Groningen, The Netherlands

**Keywords:** ACL injury, MRI, Patient specific guide, Segmentation, Reliability

## Abstract

**Background:**

Re-injury rates following reconstruction of the anterior cruciate ligament (ACL) are significant; in more than 20% of patients a rupture of the graft occurs. One of the main reasons for graft failure is malposition of the femoral tunnel. The femoral origin of the torn ACL can be hard to visualize during arthroscopy, plus many individual variation in femoral origin anatomy exists, which may lead to this malpositioning. To develop a patient specific guide that may resolve this problem, a preoperative MRI is needed to identify the patient specific femoral origin of the ACL. The issue here is that there may be a difference in the reliability of identification of the femoral footprint of the ACL on MRI between different observers with different backgrounds and level of experience. The purpose of this study was to determine the intra- and interobserver reliability of identifying the femoral footprint of the torn ACL on MRI and to compare this between orthopedic surgeons, residents in orthopedic surgery and MSK radiologists.

**Methods:**

MR images of the knee joint were collected retrospectively from 20 subjects with a confirmed rupture of the ACL. The 2D (coronal, sagittal, transversal) proton-density (PD) images were selected for the segmentation procedure to create 3D models of the femurs. The center of the femoral footprint of the ACL on 20 MRI scans, with visual feedback on 3D models (as reference) was determined twice by eight observers. The intra- and interobserver reliability of determining the center of the femoral footprint on MRI was evaluated. Intraclass correlation coefficients (ICCs) were calculated for the X, Y and Z coordinates separately and for a 3D coordinate.

**Results:**

The mean 3D distance between the first and second assessment (intraobserver reliability) was 3.82 mm. The mean 3D distance between observers (interobserver reliability) was 8.67 mm. ICCs were excellent (> 0.95), except for those between the assessments of the two MSK radiologists of the Y and Z coordinates (0.890 and 0.800 respectively). Orthopedic surgeons outscored the residents and radiologists in terms of intra- and interobserver agreement.

**Conclusion:**

Excellent intraobserver reliability was demonstrated (< 4 mm). However the results of the interobserver reliability manifested remarkably less agreement between observers (> 8 mm). An orthopedic background seems to increase both intra- and interobserver reliability. Preoperative planning of the femoral tunnel position in ACL reconstruction remains a surgical decision. Experienced orthopedic surgeons should be consulted when planning for patient specific instrumentation in ACL reconstruction.

## Background

Several factors are crucial for the success of ACL reconstruction. A surgical factor which is considered to be essential in influencing clinical outcomes is femoral tunnel placement [1, 2]. Malposition of the femoral tunnel is a risk factor for re-rupture of the graft [3]. In current surgical techniques, the location of the femoral tunnel is estimated either with a direct measurement beginning on the posterior cortex of the femur or by ‘eyeballing’ anatomical landmarks through an accessory anteromedial portal. Both techniques are profoundly dependent on the experience and preference of the orthopedic surgeon.

It is not always easy to accurately determine the exact location of the previously ruptured anterior cruciate ligament during ACL reconstruction surgery, even with the help of MR images. Artificial intelligence to aid in determination of this location is yet to be developed. A meta-analysis performed by Piefer et al. showed a wide variability in describing the femoral origin of the ACL, on radiologic as well as on arthroscopic landmarks [4]. The need for an individualized guide for optimized femoral tunnel placement seems apparent. When creating a patient specific instrument for ACL reconstruction, preoperatively a decision has to be made regarding the femoral origin of the ACL. Depending on the technique used, this point is either the starting (inside-out) or exit point (outside-in) of the drill. The aim of this study is to determine the intra- and interobserver reliability of identifying the femoral footprint of the torn anterior cruciate ligament on MRI. The influence of background (surgical or imaging) and experience of observers (surgeon or resident) is investigated.

## Methods

The research protocol met the requirements to be considered Not Human Subjects Research. This study was a retrospective study in which 20 anonymized MRI scans of subjects with a confirmed rupture of the ACL were analyzed. Scans were selected at random from a cohort of 386 chart numbers corresponding to patients over the age of 16 years, diagnosed with ACL rupture in 2018 at a university hospital. In order to be used in this study, scans had to meet the following inclusion criteria: the scan was of a subject older than 16 years of age, confirmed by closure of the distal femoral epiphysis, and the rupture of the ACL must have been confirmed by clinical diagnosis and on MRI evaluated by a medical specialist. Scans of subjects with implants, such as screws, rods, plates or knee prosthesis were excluded. Patient information, such as name, gender, age and weight, were undisclosed due to a strict anonymization protocol.

The images were acquired by a 1.5 Tesla MAGNETOM® Aera MRI scanner (Siemens Healthcare GmbH, Erlangen, Germany). The scanning protocol consisted of Proton Density series in the sagittal, coronal and axial planes. Voxel size of 0.4 × 0.4 × 3.0 mm was selected (slice thickness 3.0 mm) with a 512 × 512 matrix, a Field of View of 160x160mm, a flip angle of 150^0^, a repetition time of 3530 ms. and an echo time of 41 ms. Right knees were scanned from lateral to medial, and left knees were scanned from medial to lateral. All MRI scans were segmented to create a 3D model of the femur. Segmentation of the images was performed using Mimics (v.21, Materialise NV, Leuven, Belgium) as described by Mootanah et al. [5] Manual grey value thresholding and the Livewire tool were used in order to create the masks. Separate masks for cancellous bone, cortical bone and the overlying cartilage on MR images were combined to secure a complete model of the femur. Furthermore, manual mask adaptations were applied where necessary, such as cropping the mask and mask edges or disconnecting the femur from the tibia if the mask automatically connected them together. All the masks were converted into 3D models. To reduce artifacts from segmentation, the models were smoothed using the following parameters: smoothing factor = 0.8, number of iterations =5 and shrinking was compensated. Final femoral models were saved as a binary Standard Tessellation Language (STL) files. Creation of the 3D model took an estimated 20–30 min per case.

**Fig. 1:**
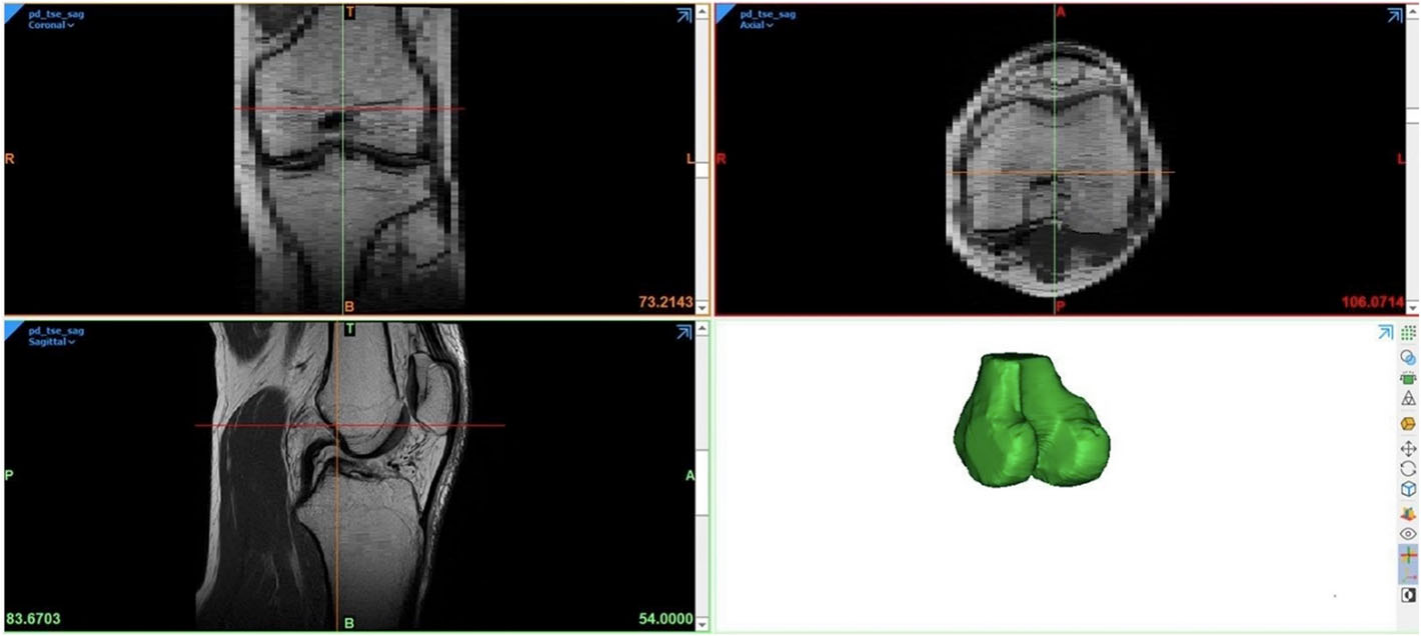
Example of the screen of the observers. The green line represents the x-axis, the orange line represents the y-axis and the red line represents the z-axis

After processing, the 2D MR Images and the 3D models were reviewed by three residents in orthopedic surgery (Res), three senior orthopedic surgeons (OS), and two fellowship trained Musculoskeletal (MSK) radiologists. The residents in orthopedic surgery were all in their fifth year of their 6 year residency program. The orthopedic surgeons had an average experience of 11 years (2, 7 and 25 years) in ACL reconstructive surgery. The MSK radiologists had an average experience of 5 years in reading MRI scans of the knee (both 5 years). None of the observers had any previous experience at identifying the femoral footprint on MRI. Observers were invited separately at the 3D laboratory of our institute. Each observer was asked to identify the center of the femoral footprint of the ACL of all 20 cases. Observers had access to the anonymized MRI and the 3D model of the femur in Mimics, an example of the screen the observers were exposed to is shown in Fig. 1. The observers could switch between a high resolution MRI image of either the sagittal, axial or coronal plane.

**Fig. 2:**
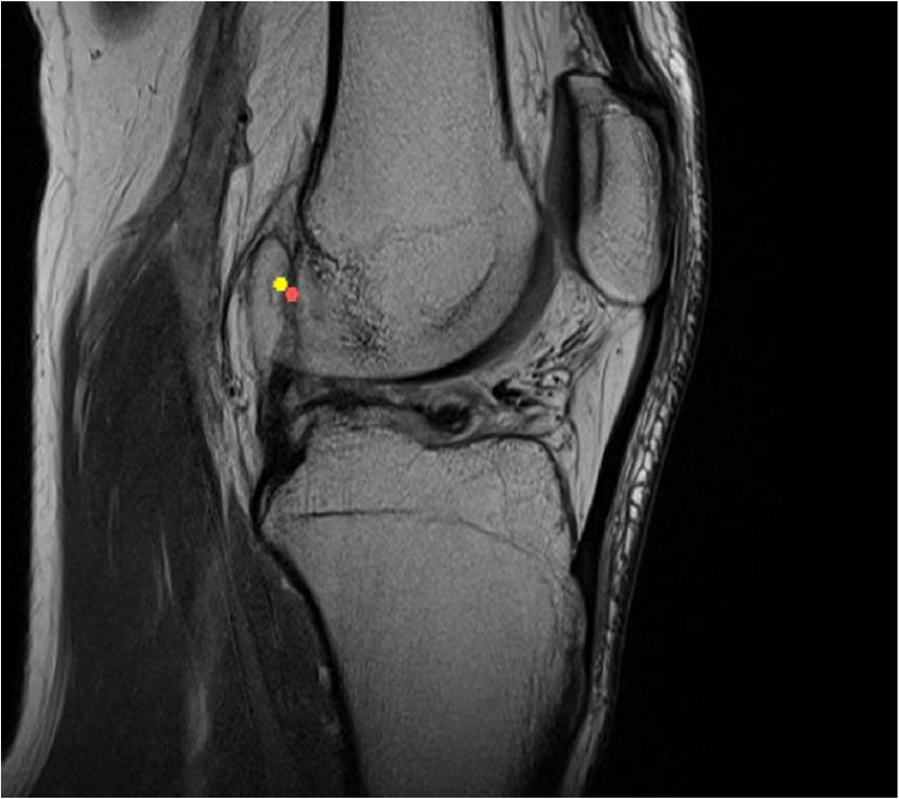
Example of the intraobserver agreement. Figure showing a sagittal slice of an MRI with two measurements from the same observer with at least one week interval

Using the Mimics software, observers were asked to place a circle of 8 pixels in diameter on a sagittal MRI image of their choice, with the other planes and 3D model as a reference, at the center of the patient specific femoral footprint of the ACL. An example is shown in Fig 2. After approximately 1 week the procedure was repeated by the same observers. All observers were blinded to the results of their first session and those of the other observers. As the observers were not trained in Mimics, a medical student trained in Mimics was present at both sessions for practical questions and to ensure smooth logistics.

### Data processing and statistical analysis

The x, y, z coordinates were calculated for each of the marked points using Statistical Package for the Social Sciences version 23 (SPSS, IBM, Armonk, NY, US). The center of the scan was automatically defined as 0. The x-axis represents the lateral-to-medial direction, running from left to right in both left and right knees, the y-axis the anterior-to-posterior direction and the z-axis the caudal-to-cranial direction. To quantify the intra- and interobserver reliability, the distance between the first and the second assessment and the distance between observers was calculated for each coordinate and the 3D point (i.e. x, y, z and 3D). The total 3D distance between the marked points was calculated using the following formula, where × 1, y1 and z1 represents observer 1 or measurement 1 and × 2, y2 and z2 represents observer 2 or measurement 2.

$$ Dtotal=\surd {\left(x1-x2\right)}^2+{\left(y1-y2\right)}^2+{\left(z1-z2\right)}^2 $$

Intraclass correlation coefficients (ICC 2-way random, absolute agreement) were calculated between the first and second assessment of an observer and between observer groups (Orthopedic Residents, Orthopedic Surgeons and MSK radiologists). Values less than 0.5 were considered to be indicative of poor reliability, values between 0.5 and 0.75 indicate moderate reliability, values between 0.75 and 0.9 indicate good reliability, and values greater than 0.90 indicate excellent reliability [6]. Scatter plots using the Bland & Altman method were used to visually assess agreement between raters [7, 8]. This was performed for the X, Y, Z and 3D coordinate. The mean difference and the limits of agreement were calculated and depicted in the scatter plots. Statistical analyses were performed in close collaboration with a biostatistician.

## Results

**Fig. 3:**
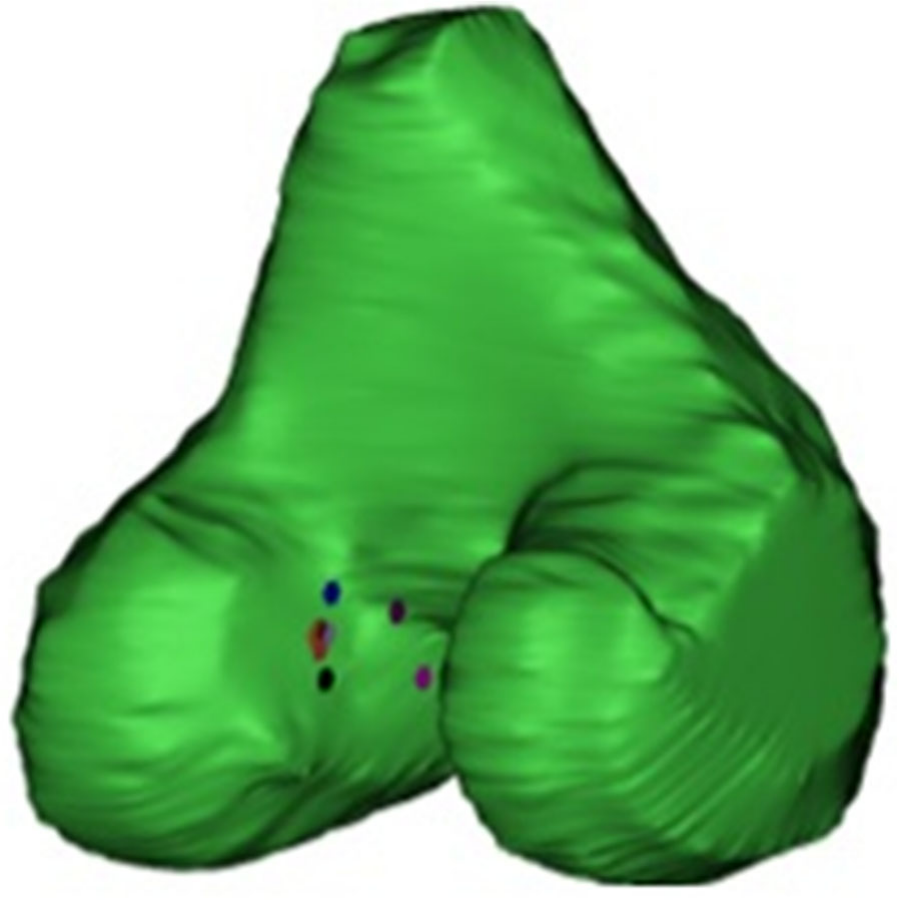
Example of marker points of all observers combined in one case: Orthopedic residents (purple, orange and violet), orthopedic specialists (red, wine and black) and MSK radiologists (pink and blue)

The 3D-femur models in Fig. 3 illustrate the observer’s scattered marker points.

The absolute mean differences between two measurements regarding the x, y, z and 3D-coordinates as well as the result from the ICC calculations are depicted in Table 1. All mean differences per coordinate between the first and second session were below 2.78 mm. The mean 3D distances per group were 3.47 mm, 2.97 and 5.21 mm for the Res, OS and MSK group respectively.

**Table 1:** Intraobserver reliability per coordinate and in total: differences between the 1st and 2nd session. Distances are depicted in mm

Observer	X coordinate		Y coordinate		Z coordinate		3D Distance
	Mean difference (SD)	ICC	Mean difference (SD)	ICC	Mean difference (SD)	ICC	Mean difference (SD)
Res-1	0.27 (1.77)	1.000	0.00 (2.06)	0.994	1.65 (1.78)	0.987	3.03 (1.95)
Res-2	0.58 (2.40)	0.999	2.12 (2.35)	0.984	1.34 (3.02)	0.979	4.58 (2.29)
Res-3	0.29 (1.60)	1.000	0.13 (2.70)	0.989	1.99 (1.91)	0.986	3.62 (2.00)
OS-1	0.51 (1.20)	1.000	1.28(1.54)	0.994	0.63 (1.97)	0.992	2.74 (1.49)
OS-2	0.13 (1.74)	1.000	1.03 (2.01)	0.993	1.52 (2.37)	0.985	3.55 (1.73)
OS-3	0.31 (2.70)	0.999	1.42 (1.43)	0.993	0.07 (1.33)	0.997	2.63 (2.45)
MSK-1	0.58 (4.11)	0.998	2.26 (3.19)	0.976	2.78 (3.51)	0.961	6.29 (3.41)
MSK-2	0.18 (1.89)	0.999	1.24 (2.19)	0.990	2.05 (3.17)	0.969	4.13 (2.52)
Total	0.35	n.a.	1.15	n.a.	1.50	n.a.	3.82

*Res* resident orthopedic surgery, *OS* senior orthopedic surgeon, *MSK* fellowship trained musculoskeletal radiologist, *SD* Standard Deviation, *ICC* intraclass correlation coefficient, *n.a.* not applicable. The x-axis represents the lateral-to-medial direction, the y-axis the anterior-to-posterior direction and the z-axis the caudal-to-cranial direction

Table 2 shows the interobserver reliability between groups and show excellent ICC values between groups (ICC > 0.95). Table 3 shows the interobserver reliability within the groups. Also excellent ICC values were shown within the OS and RES groups (ICC < 0.95). The MSK group shows good results. While the agreement regarding the x-coordinate was excellent (ICC > 0.95), the agreement regarding the y and z-coordinate were good (ICCs 0.890 and 0.800, respectively). Table 4 shows the mean 3D distances in millimeters between the first and second assessment, as well as the mean difference in 3D distance between the observers per group.

**Table 2:** Interobserver reliability between groups per coordinate

	ICC X coordinate	ICC Y coordinate	ICC Z coordinate
Res vs MSK	0.999	0.970	0.953
Res vs OS	0.999	0.960	0.952
OS vs MSK	1.000	0.982	0.987

*Res* resident orthopedic surgery, *OS* senior orthopedic surgeon, *MSK* fellowship trained musculoskeletal radiologist, *ICC* intraclass correlation coefficient. The x-axis represents the lateral-to-medial direction, the y-axis the anterior-to-posterior direction and the z-axis the caudal-to-cranial direction

**Table 3:** Interobserver reliability within groups per coordinate

Group	ICC X-coordinate	ICC Y- coordinate	ICC Z-coordinate
Res	0.999	0.962	0.982
OS	1.000	0.995	0.961
MSK	0.998	0.890	0.800

*Res* resident orthopedic surgery, *OS* senior orthopedic surgeon, *MSK* fellowship trained musculoskeletal radiologist, *ICC* intraclass correlation coefficient. The x-axis represents the lateral-to-medial direction, the y-axis the anterior-to-posterior direction and the z-axis the caudal-to-cranial direction

**Table 4:** Mean 3D distance difference in mm per group

Group	Mean 3D distance difference between first and second assessment	Mean 3D distance difference between the observers
Res	3.74 mm	6.57 mm
OS	2.97 mm	5.62 mm
MSK	5.21 mm	13.64 mm
All	3.82 mm	8.67 mm

*Res* resident orthopedic surgery, *OS* senior orthopedic surgeon, *MSK* fellowship trained musculoskeletal radiologist, *All* all observers, *ICC* intraclass correlation coefficient, *mm* millimeter

**Fig. 4:**
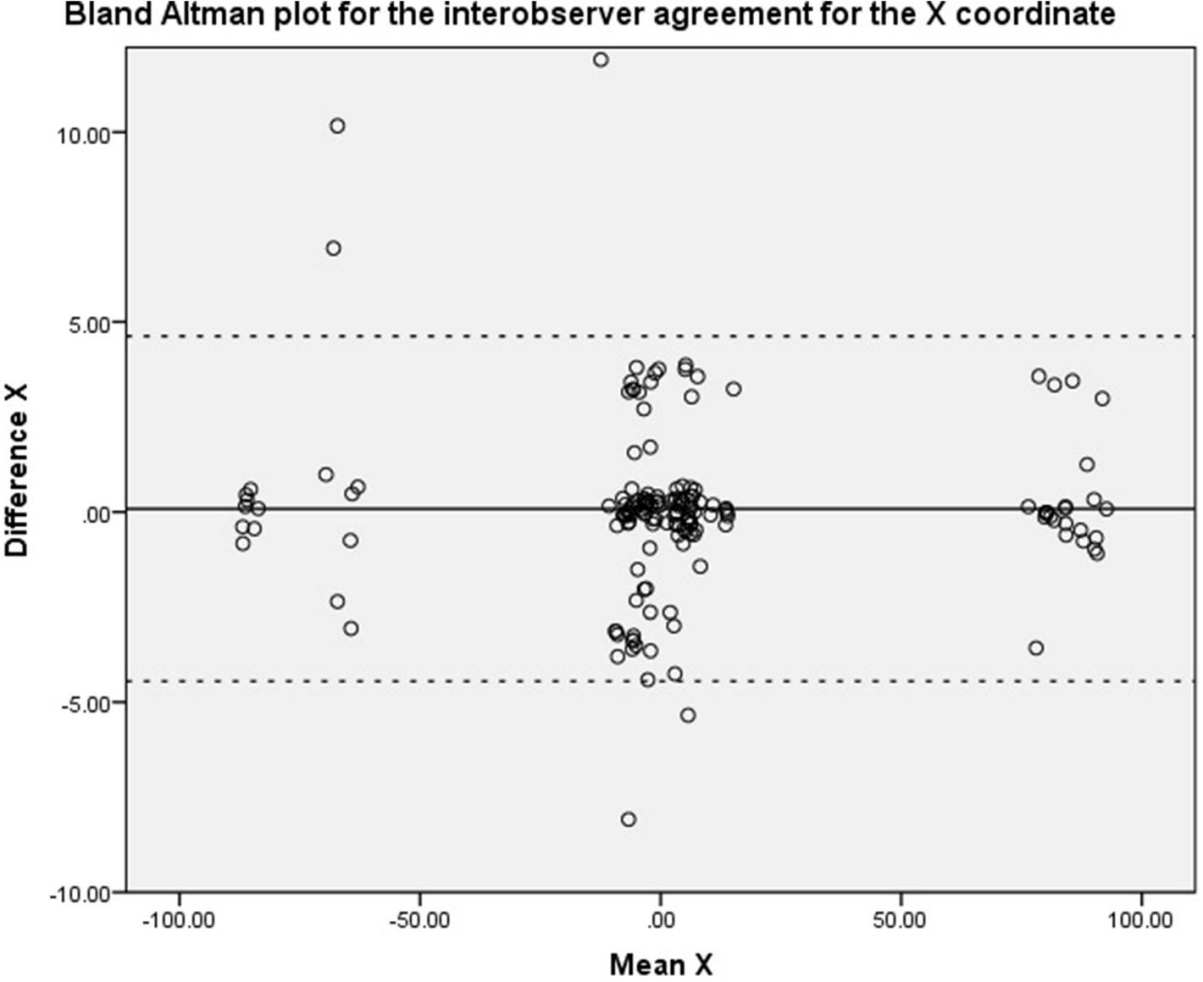
Bland & Altman scatter plot for the X coordinate. Solid black line refers to the mean difference, dashed line illustrates the upper and lower bound of the 95% confidence interval of the difference. Values represent the number of pixels in which 0 represents the center of the scan

**Fig. 5:**
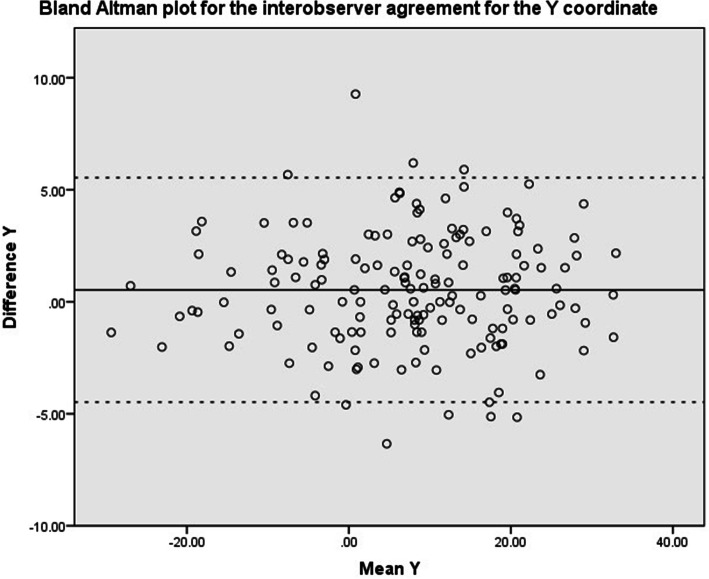
Bland & Altman scatter plot for the Y coordinate. Solid black line refers to the mean difference, dashed line illustrates the upper and lower bound of the 95% confidence interval of the difference. Values represent number of pixels in which 0 represents the center of the scan

**Fig. 6:**
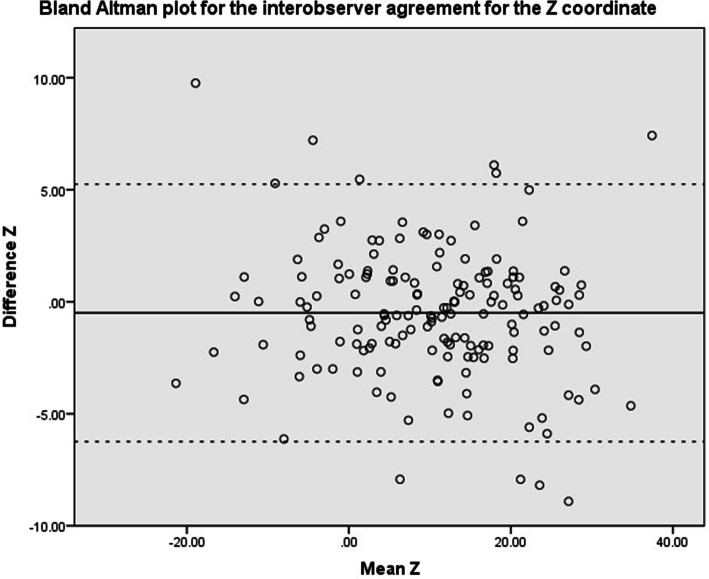
Bland & Altman scatter plot for the Z coordinate. Solid black line refers to the mean difference, dashed line illustrates the upper and lower bound of the 95% confidence interval of the difference. Values represent number of pixels in which 0 represents the center of the scan

**Fig. 7:**
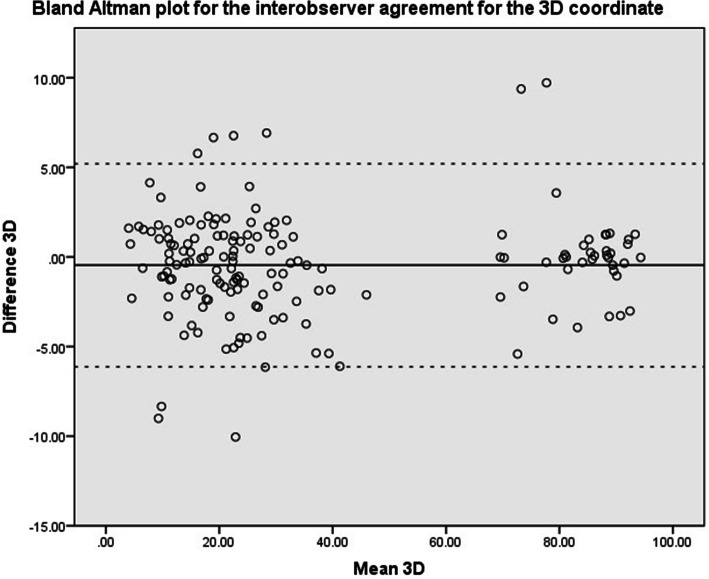
Bland & Altman scatter plot for the 3D coordinate. Solid black line refers to the mean difference, dashed line illustrates the upper and lower bound of the 95% confidence interval of the difference. Values represent number of pixels in which 0 represents the center of the scan

Scatter plots of the Bland & Altman methods are shown in Fig. 4, Fig. 5, Fig. 6 and Fig. 7 for the X, Y, Z and 3D coordinate respectively. These plots illustrate the absence of a systematic bias between measurements. A trimodal distribution is observed for the X coordinate due to the fact that the database includes scans of both left and right knees. In left knees, the medial wall of the lateral femoral condyle will be located in positive X coordinate ranges, whereas in right knees, the same location will be in negative X values. The trimodal distribution as seen in Fig. 4 is representation of slice thickness which lead to a stepwise increment of values for the X coordinate.

The 3D coordinate only reads positive values, as a consequence of the formula used to calculate the 3D coordinate. As seen in the methods section, a square root is taken, which results in positive values. Therefore the trimodal distribution is transformed into a bimodal distribution.

## Discussion

To our knowledge, this is the first study to evaluate the intra- and interobserver reliability when determining the femoral ACL attachment site in full-grown ACL deficient subjects on MRI. Excellent intraobserver and interobserver reliability is shown. Orthopedic surgeons with experience in ACL reconstruction were most consistent with a mean difference of 2.97 mm between the first and second assessment. The assessments of the x-coordinate showed an excellent agreement, while those on the y- and z-coordinates showed slightly lower ICC values, but still classify as excellent agreement. This miniscule difference, although not significant, could be explained by the anterior-to-posterior and caudal-to-cranial planes compared to the lateral-to-medial plane. This implies that nearly all observers selected the same sagittal slice to identify the center of the femoral footprint of the ACL. The challenge in identifying the femoral footprint of the ACL is how deep and/or how high the footprint is located on the medial wall of the lateral femoral condyle, hence the anterior-to-posterior and caudal-to-cranial planes (y-coordinate and z-coordinate respectively). This seems to be reflected in the found ICCs.

Our results are in contrast to those of Swami et al., who studied 62 MRI scans in pediatric patients, half of which contained an ACL tear [9]. A mean intraobserver difference of 1.2 mm (± 0.7 mm) and a mean interobserver difference of 1.8 mm (±1.1 mm) were shown. Swami et al. asked their observers, which included one radiologist and one medical student, to identify the entire geometry of the footprint of the ACL, out of which a center point was calculated and used for comparison [9]. The geometry of the femoral insertion of the ACL compromises approximately 100mm^2^ (50-197 mm^2^ reported) [10–12]. In our study, observers were asked to identify the center of the footprint with a small circle of only 8 pixels, replicating a Kirschner wire in the center of the stump of the torn ACL. Identifying a surface from which a center point is calculated may be more forgiving than direct determination of a center point which can explain the difference in results between our study and the results of Swami et al. On the other hand, asking an observer to determine the center of the femoral footprint can be regarded as a more complex task compared to drawing the entire geometry of the femoral footprint of the ACL. When an observer is asked to identify a center of an ellipse, one first has to define the ellipse in his mind. This potentially decreased the reliability as a consequence of the methods used in our study, but still excellent reliability is demonstrated.

Swami studied pediatric patients ranging from 10 to 17 years of age [9]. Our study only included scans of subject with closed epiphysis of the distal femur, which implies subjects were over 16.6 years of age [13]. The exact age and sex distribution among our subjects could not be retrieved due to a strict anonymization protocol. The presence of open epiphysis can influence the choice of treatment in ACL injury [14]. Whether the age of the subjects influences an observer’s performance to determine the femoral footprint of the ACL is unknown.

Our findings are comparable to the findings from Rachmat et al. who demonstrated a mean intraobserver accuracy of 4.30 mm when identifying the femoral footprint of the ACL on MRI [15]. It has to be noted however that Rachmat et al. used one cadaveric specimen with an intact ACL. Our study is thus more representative of the clinically relevant situation. Adding MRI’s of subjects with intact ACL’s to our database could have introduced a learning effect with the observers. The effect of background and experience may then have been biased. Therefore our study only included MRI with confirmed rupture of the ACL.

In our study orthopedic surgeons were able to determine the same point (femoral footprint) with a mean difference of 2.97 mm between two assessments of 20 scans. A high diversity in the size and shape of the femoral footprint has been reported [12], and this footprint appears to be ribbon shaped with a length of 16 mm and a width of 8 mm [11, 12]. In this light, a mean 3D difference of 2–5 mm can be regarded acceptable.

The orthopedic surgeons showed the highest agreement within their group compared to the other two groups, followed by the orthopedic residents. These findings seem to indicate a lack of “in-field” experience of radiologists compared to the orthopedic surgeons and orthopedic residents. Possibly, witnessing or performing an ACL reconstruction (or knee surgery in general) repeatedly, leads to more consistency in defining the location of the ACL footprint. As residents in orthopedic surgery, not specialized in ACL reconstruction, attained comparable group interobserver reliability compared to the orthopedic surgeons, the effect of general surgical experience seems to be more relevant than experience in ACL reconstruction specifically. This emphasizes that femoral tunnel positioning remains a surgical decision, although it may not always has to be taken in the operating theatre.

The excellent ICC values mainly show that the observers are consistent with locating the same point. It may seem tempting to compare the ACL insertion points as determined by the observers to a predefined point measured from the posterior cortex, for instance as defined by Piefer [4]. This would not be in accordance with the patient specific (instrumentation) concept and would lead to a generalized approach for each patient. No gold standard, such as confirmation by arthrotomy, was used in this study to prove that this point is actually the femoral insertion of the ACL. This is due to the fact that scans of patients with torn ACL’s were used and not cadaver samples. The down side of using cadavers is the intactness of the ACL. The ultimate goal of identifying the femoral insertion of the ACL is to give the surgeon the optimal information about where the femoral tunnel should be located. This is, obviously, only necessary in case of a torn ACL. Therefore for clinical purposes, this study was set up to use scans of a cohort of patients resembling the relevant population.

As a consequence, we included subjects who have undergone routing 2D MRI scans of the knee for clinical purposes. It has been shown that 3D MRI improves overall image quality and quantitative contrast ratio [16], although it has not been more accurate in diagnosing ligamentous injuries compared to 2D MRI [17]. It has been demonstrated that there is no advantage in localizing the ACL attachment centers when using 3D MRI over 2D MRI [10].

In our study manual segmentation of the MRI scans was performed to create a 3D model of the distal femur. Automatic or semi-automatic segmentation techniques have been described in the literature [18–20]. Although the correctness of the 3D model was not evaluated in this study, evaluation of the segmentation technique was done prior to this study. Unpublished data showed an excellent surface comparison when comparing 3D models derived from 2D MRI, 3D MRI and CT.

The fact that orthopedic surgeons reach a high group interobserver agreement may be the effect of a monocenter study. There may be a consensus on femoral tunnel position within a group of direct colleagues. Furthermore, this consensus is transferred to the orthopedic residents during their training. A multicenter and possibly even a multinational study would be needed to determine if this is indeed the case.

## Conclusion

The aim of this study was to determine the intra- and interobserver reliability of identifying the femoral footprint of the anterior cruciate ligament on MRI. Excellent intraobserver agreement was demonstrated. The interobserver reliability was less compared to the intraobserver reliability. Orthopedic surgeons had a higher level of intra- and interobserver agreement compared to MSK fellowship trained radiologists and, to a lesser extent, to residents in orthopedic surgery. Employing this feature, experienced orthopedic surgeons are the preferred physicians to preoperatively plan femoral tunnel positioning in patient specific ACL reconstruction. By doing so, femoral tunnel malposition may become less of a problem in ACL reconstruction, increasing return to play rates and decreasing re-rupture rates following ACL reconstruction.

## Data Availability

The datasets used and analyzed during the current study are available from the corresponding author on reasonable request.

## Abbreviations

2D: 2 dimensional
3D: 3 dimensional
ACL: Anterior Cruciate Ligament
ICC: Intraclass correlation coefficient
mm: millimeter
ms: millisecond
MRI: Magnetic Resonance Imaging
MSK: Musculoskeletal
OS: Orthopedic Surgeon
Res: Resident Orthopedic Surgery
STL: Standard Tessellation Language
